# Mapping Stem Rust (*Puccinia graminis* f. sp. *secalis*) Resistance in Self-Fertile Winter Rye Populations

**DOI:** 10.3389/fpls.2020.00667

**Published:** 2020-05-26

**Authors:** Paul Gruner, Anne-Kristin Schmitt, Kerstin Flath, Brigitta Schmiedchen, Jakob Eifler, Andres Gordillo, Malthe Schmidt, Viktor Korzun, Franz-Joachim Fromme, Dörthe Siekmann, Anna Tratwal, Jakub Danielewicz, Marek Korbas, Karol Marciniak, Renata Krysztofik, Małgorzata Niewińska, Silvia Koch, Hans-Peter Piepho, Thomas Miedaner

**Affiliations:** ^1^State Plant Breeding Institute, University of Hohenheim, Stuttgart, Germany; ^2^Institute for Plant Protection in Field Crops and Grassland, Julius-Kuehn Institute, Kleinmachnow, Germany; ^3^KWS LOCHOW GmbH, Bergen, Germany; ^4^KWS SAAT SE & Co. KGaA, Einbeck, Germany; ^5^Federal State Budgetary Institution of Science Federal Research Center “Kazan Scientific Center of Russian Academy of Sciences”, Kazan, Russia; ^6^HYBRO Saatzucht GmbH & Co. KG, Schenkenberg, Germany; ^7^Institute of Plant Protection – National Research Institute, Poznań, Poland; ^8^Danko Hodowla Roślin Sp. z o.o., Kościan, Poland; ^9^Biostatistics Unit, Institute of Crop Science, University of Hohenheim, Stuttgart, Germany

**Keywords:** rust, all-stage resistance, adult-plant resistance, QTL, mapping, mixed model, leaf rust, hybrid rye

## Abstract

Rye stem rust caused by *Puccinia graminis* f. sp. *secalis* can be found in all European rye growing regions. When the summers are warm and dry, the disease can cause severe yield losses over large areas. To date only little research was done in Europe to trigger resistance breeding. To our knowledge, all varieties currently registered in Germany are susceptible. In this study, three biparental populations of inbred lines and one testcross population developed for mapping resistance were investigated. Over 2 years, 68–70 genotypes per population were tested, each in three locations. Combining the phenotypic data with genotyping results of a custom 10k Infinium iSelect single nucleotide polymorphism (SNP) array, we identified both quantitatively inherited adult plant resistance and monogenic all-stage resistance. A single resistance gene, *tentatively named Pgs1*, located at the distal end of chromosome 7R, could be identified in two independently developed populations. With high probability, it is closely linked to a nucleotide-binding leucine-rich repeat (NB-LRR) resistance gene homolog. A marker for a competitive allele-specific polymerase chain reaction (KASP) genotyping assay was designed that could explain 73 and 97% of the genetic variance in each of both populations, respectively. Additional investigation of naturally occurring rye leaf rust (caused by *Puccinia recondita* ROEBERGE) revealed a gene complex on chromosome 7R. The gene *Pgs1* and further identified quantitative trait loci (QTL) have high potential to be used for breeding stem rust resistant rye.

## Introduction

Rye stem rust caused by *Puccinia graminis* f. sp. *secalis* ERIKSS. & HENNING can be a severe threat for rye (*Secale cereale* L.) in epidemic years. After the fungus has entered the plants in early summer, it produces high amounts of urediniospores that subsequently infect new plants and spread the disease over large areas. Infected plants show spore-filled cracks all over the stems, and if the infection level is high, even on leaves and heads. As a result, water and nutrient transport breaks down and grain yield loss can reach levels of 60% (Solodukhina and Kobylyansky, 2001). Yield losses due to rye stem rust are reported from Northeastern Europe, the largest growing region worldwide, but the disease was also reported to occur in Brazil (Roelfs, 1985) and South Africa (Boshoff et al., 2019).

Rye is an outcrossing species and consequently the first rye varieties were populations composed of self-incompatible and genetically differing plants. In Germany, hybrid rye breeding started in 1971. In contrast to population breeding, hybrid breeding is based on the combination of inbred lines carrying a self-fertility gene. Heterotic groups and a cytoplasmic-genic male sterility system are used to exploit heterosis and to facilitate seed production on a large scale (Geiger and Miedaner, 2009). To select for grain yield and other heterotic traits, testcrosses are produced by crossing inbred lines to single-cross testers of the opposite heterotic group and by evaluating their testcross performance in the year after.

Today, there are no newly developed rye cultivars in Germany registered that are known to carry stem rust resistances. Genetic resources having different levels of resistance were reported as old population varieties from Russia, Hungary, and Argentina, Austrian landraces and US-fodder rye (Miedaner et al., 2016). Additionally, Solodukhina and Kobylyansky (2001) reported resistant plants in populations from Italy, China, Sweden, Uruguay, the Czech Republic, Azerbaijan, former Yugoslavia, Lithuania, Ukraine, Bulgaria, Portugal, Finland, and Great Britain. Moreover, Boshoff et al. (2019) found resistant plants in South African fodder rye.

Detailed genetic studies by Tan et al. (1976, 1977) indicate that there are several resistance genes in the same rye population. Inbred lines developed from various populations were crossed and the offspring was investigated. They used forma specialis *secalis* and forma specialis *tritici* for inoculation and identified six and eight resistance genes for each forma specialis, respectively. Resistance donors were the varieties “Kenya” from Kenya, “Wrens” and “Gator” from the United States and the cross “Elbon × Gator.” Solodukhina and Kobylyansky (2007) found two additional resistance genes of which one was present in two Russian population varieties named “Kharkovskaya” and “Rossul” (Solodukhina and Kobylyansky, 2000). The majority of the genes were reported to act dominantly and to be race-specific (Tan et al., 1976, 1977; Solodukhina and Kobylyansky, 2007). Further gene actions like partial dominance, recessiveness or epistasis were discussed (Tan et al., 1976, 1977), and rye populations without hypersensitive reactions, but sparsely dispersed pustules pointing to quantitative resistance were observed (Solodukhina and Kobylyansky, 2001).

Most of the studies on stem rust resistance also investigated rye leaf rust (caused by *Puccinia recondita*) resistance (Solodukhina and Kobylyansky, 2001, 2007) or were based on material known to contain leaf rust resistances (Morey, 1959; Tan et al., 1976, 1977). Leaf rust in rye is naturally occurring in all rye growing regions (Wehling et al., 2003). Being both race-specific or non-race-specific, resistance genes were found on all rye chromosomes except chromosome 5R (Wilde et al., 2006). Tight linkage or pleiotropy is not evident for resistances to different rusts and other pathogens in rye. In wheat, however, there are several examples that show pleiotropy or tight linkage of resistance genes to several rusts (Seah et al., 2001; Krattinger et al., 2009; Singh et al., 2011).

A lot of effort was put into the investigation of stem rust resistance in wheat. McIntosh et al. (2013, 1975) listed 59 resistance genes plus several temporarily designated genes for stem rust in wheat and the gene function of a further one, *Sr60*, has recently been described (Chen et al., 2020). Some of these resistance genes were derived from the rye genome, e.g., *Sr27*, *Sr1R*^*Amigo*^, *Sr50* and *Sr31* (Mago et al., 2015; Rahmatov et al., 2016). The latter protected wheat from stem rust infections for decades until the emergence of the Ug99 lineage (Singh et al., 2011). By using transgenic methods, the *Sr50* gene coding for a coiled-coil nucleotide-binding leucine-rich repeat (NB-LRR) protein was successfully transformed into a susceptible wheat line (Mago et al., 2015). The tight genetic relation of wheat and rye is not only restricted to the host side of the pathosystem. The two stem rust pathogen formae speciales of rye (*secalis*) and wheat (*tritici)* can hybridize and, like wheat and rye, probably also had a common ancestor (Johnson et al., 1932; Martis et al., 2013).

QTL mapping based on the linkage associated with biparental populations proved to be a solid tool so that special software like plabqtl (Utz, 2012) or Rqtl (Broman et al., 2003) is still commonly used. Our study was not based on these routines, because of two main reasons. Firstly, as those methods make use of pre-calculated means it is difficult to implement error structure into the analysis. Secondly, the procedures are based on interval mapping methods (Lander and Botstein, 1989; Jansen, 1993) where dense marker maps are reduced for analysis, often to predefined cM distances. As we were interested in the most significant marker and had high enough marker densities for single-point analysis, interval mapping was considered unnecessary. If the most promising markers from a single nucleotide polymorphism (SNP) array are identified, they can easily be transferred into a competitive allele-specific polymerase chain reaction (KASP) genotyping assay, which is much more cost efficient for single markers. With the aim of a proper modeling of both, phenotypic data and the association of phenotypic and genetic data, we decided to use linear mixed models. This method was approved by working groups with high statistical expertise, for example by Piepho (2000, 2005) and Malosetti et al. (2011). The modeling allowed us to account for the unbalanced and complex experimental design and by further model extension to test significance of markers. Even further, the mixed model for a single trait can be extended into a bivariate or multivariate form. It allows calculation of genetic correlation for lines and respective testcross performance but also for two traits measured in the same plot. Estimated covariance for factors can increase the power of the model fits. Like a QTL mapping approach with single-trait mixed models, the bivariate models can also be extended by marker effects to test for significant gene loci. Jiang and Zeng (1995) showed in a linkage mapping approach that different models defined by different hypotheses can be used to test linkage vs. pleiotropic QTL effects with a special focus on common QTL positions for two traits. Similar approaches based on mixed model theory have also been applied for genome-wide association studies (Segura et al., 2012).

Most of the studies in wheat focus on resistance testing with seedlings (McIntosh et al., 1995). Seedling resistances genes are active in the seedling and adult-plant stage, so that the term all-stage resistance (ASR) is often used synonymously (Ellis et al., 2014). Adult-plant resistance (APR) on the other hand cannot be identified in the seedling stage and was described less frequently for wheat stem rust (McIntosh et al., 1995). To detect APR too, our study was based on field tests. Additionally, a leaf-segment test (LST, seedling test) of the parental lines was used to confirm whether the resistance is also active in the seedling stage.

To our knowledge, no molecular-genetic studies about stem rust resistance in rye, i.e., about markers linked resistance loci, have been published, but QTL mapping studies in rye on leaf rust resistances (Wehling et al., 2003; Roux et al., 2004) and agronomic traits (Miedaner et al., 2012; Hackauf et al., 2017) were established. Our objectives were: (1) application of mixed models for modeling unbalanced field data and single point QTL mapping, (2) identification of resistance genes and QTL in three bi-parental populations of inbred lines derived from a cross of the type “susceptible × resistant,” (3) differentiation between ASR by LST in seedling stage with parental lines and some of the most resistant progeny and APR in the field, (4) comparison of line per se performance in relation to testcross performance with application to QTL mapping, and (5) investigation of LR resistance with special focus on the detection of pleiotropic or linked genes of LR and SR resistances.

All progenies of the populations were tested in artificially inoculated field trials over 2 years and three locations each. An additional testcross population was developed from one of the bi-parental populations and tested accordingly. Genotyping was conducted using a 10k SNP chip and the most promising markers linked with resistance loci were retested with a KASP assay.

## Materials and Methods

### Mapping Populations

Three mapping populations (P1, P2, P4) with 68 to 70 progenies each were analyzed in this study. P1 and P2 were developed by crossing a self-fertile and SR-susceptible breeding line with a SR resistance donor. The resistance donors for P1 and P2 were self-fertile lines previously developed from genetic resources listed in Table 1. From the F_2_-generation, P1 and P2 were further propagated by single-seed descent to produce recombinant inbred lines. The inbreeding level of the respective populations can be found in Table 1. To create P4, one single self-incompatible (i.e., non-self-fertile) plant from a SR resistance-tested full-sib family was crossed with a self-fertile breeding line (L403). Self-fertility is dominantly inherited, so that the resulting F_1_ generation could be self-pollinated. Because we did not know whether the single donor plant of P4 was homozygously resistant, several crosses were performed and those selected where the F_1_ was not segregating for stem rust. To increase the amount of genes from the elite line (L403), a F_1_ plant was backcrossed with L403. The resulting BC_1_F_1_ plant was self-pollinated like P1 and P2. Because the resistance donor of P4 was one single self-incompatible plant, it could not be further maintained and consequently was not part of the experiments. Additionally, all inbred lines from P1 were crossed with a SR-susceptible single cross tester (developed by and being proprietary to KWS LOCHOW GmbH) and the resulting three-way hybrids were also tested in the field (P1TC).

**TABLE 1:** Size, parents, origin, and generation of self-pollination (selfing generation) of the mapping populations.

**Population**	**Susceptible parent**	**Source of resistance donor^a^**	**Selfing generation**	**Number of genotypes**	**Breeder/Institute^b^**
P1	KWL1770_90	Russia/NEM LN461	F_2:4_	68	KWL
P1TC	KWL1770_90	Russia/NEM LN461	(F_2:__3_) × T	68	KWL
P2	KWL1770_90	Russia/VIR818	F_2:3_	68	KWL
P4	L403	Russia/NEM Hy 75/81	BC_1_F_2:3_	70	UHOH

*^*a*^VIR = N. I. Vavilov All-Russian Institute of Plant Genetic Resources, Saint Petersburg, Russia; NEM = Research and Development Institute of Agriculture of Central Regions of Non-Chernozem Zone of the Russian Federation in Nemchinovka near Moscow, Russia, ^*b*^KWL = KWS LOCHOW GmbH, Bergen, Germany; UHOH = University of Hohenheim, Stuttgart, Germany.*

Resistance donors (Table 1) were “VIR 818” (P2), a genetic resource from the Vavilov All-Russian Institute of Plant Genetic Resources in Saint Petersburg, Russia (O.V. Solodukhina) and two donors, “NEM LN461” (P1) and “HY75/81” (P4), that were improved for rust resistances and have been received from the Research and Development Institute of Agriculture of Central Regions of Non-Chernozem Zone of the Russian Federation (A. A. Goncharenko) in Nemchinovka near Moscow in Russia.

### Field Trials

Two-year field trials with scorings in 2017 and 2018 were conducted in Poland in Koscielna Wies (KOS) and in Germany in Stuttgart-Hohenheim (HOH), Berlin-Dahlem (DAH), Petkus near Baruth/Mark (PET) and Wohlde near Bergen (WOH), in a total of ten environments (5 sites × 2 years). Additionally, 70 breeding lines (B1, B2, B3) and a further bi-parental population with 72 genotypes (P3) were grown. Twenty-five breeding lines (B1) were overlapping between all locations (Table 2). Both, the single populations (P1–P4, P1TC) and the additional breeding lines (B1–B3) were each denominated as a single set. All genotypes from a single set were randomized in an alpha-design with two replicates per genotype and tested in three of the locations per year (Table 2). For each population the parental genotypes were added twice each. At each site, sets were adjacent to each other. The same allocation of material was used for both years. Population P3 was excluded after the first analysis of variance because it revealed no significant genotypic variance. Field entries (plots) were single rows of 1 to 1.5-m length separated to the neighboring plot by susceptible “spreader” lines (pre-tested breeding line), serving to further spread the disease after inoculation and reduce neighboring effects of field entries. About 50 to 60 seeds were sown per row in the autumn of the year before disease assessments.

**TABLE 2:** Number of genotypes and their allocation in 2017 and 2018.

**Set/Pop**	**HOH**	**DAH**	**KOS**	**PET**	**WOH**
P1	72	.	.	72	72
P1TC	72	.	.	72	72
P2	.	72	.	72	72
P3	.	.	72	72	72
P4	72	72	72	.	.
B1	25	25	25	25	25
B2	25	25	25	.	.
B3	20	20	20	.	.

*German locations were: HOH = Stuttgart-Hohenheim; DAH = Berlin-Dahlem; PET = Petkus, WOH = Wohlde; Polish location was: KOS = Koscielna Wies.*

### Spore Production, Inoculation, and Trait Assessment

Details about spore production and inoculation procedure can be found in Miedaner et al. (2016). In short, a mixture of three stem rust isolates was used for inoculation. It was based on previous collections in naturally infected sites in Germany and Poland. The isolates were selected to possess different virulence combinations and to represent the German stem rust population as determined in a previous project. To have pure strains and to distinguish different races, urediniospores from a single pustule were multiplied (single-pustule isolate) on leaves of susceptible rye seedlings (cultivar “Palazzo”). The pathogen race was determined by individually inoculating each culture on a differential set of 15 rye lines with different (combinations of unknown) resistance genes (Supplementary Table S1, Miedaner et al., 2016). High quantity of spores could be produced on rye seedlings (cultivar “Palazzo”) without detaching the leaves. The dates of inoculation were chosen on weather conditions and developmental stage of the plants. In the interval from mid-heading of plants until end of flowering (BBCH 55-65), all plots in all environments were sprayed two times with 5–10 days in between by an urediniospore-agar suspension (120 mg of spores per 100 m^2^) using a spinning disk sprayer (Micron Ulva, Bromyard Industrial Estate, Bromyard, Herefordshire HR7 4HS, United Kingdom). Starting with the first visually distinguishable symptoms between the plots (beginning/mid of kernel ripening, BBCH 80-84), the percentage of stem surface between the second leaf (F-1) and the node above covered with urediniospores was visually assessed (0–100%). For a single score, stems from all plants in the plot were considered, so that an average score for a plot was given. Rating was repeated three times at intervals of about 1 week.

In all years, the additional traits heading date (HD), in days from first of January, and plant height (PH), measured as the distance from ground to the top of the ear, were evaluated. In all locations in the second year (2018), also a rating for naturally occurring leaf rust (LR) was conducted for all populations. The percentage of leaf area (first leaves below the flag leaf) covered with spores was assessed plotwise and was available for all bi-parental populations.

### Leaf-Segment Test

The LST was used to distinguish between APR and ASR. The parents and selected genotypes from the progenies were tested with six SR isolates differing in their reaction to the differential lines and including the three isolates used for field inoculation (Supplementary Table S1). Eight to 15 plants per single isolate and genotype were tested. Leaves from 10-days-old seedlings were placed in petri dishes filled with agar plus benzimidazole (35 mg kg^–1^) and silver nitrate (1.5 mg kg^–1^). After inoculation with urediniospores by a cotton swab, the petri dishes were placed for 24 h in dark chambers at 20°C with 100% humidity followed by 14 days of permanent light. The infection type was scored following a modified scale from Stakman et al. (1962), so that no subgroups were allowed (“0′” = “0;” = “;” = 0; “1′” = 1; “2′” = 2; “3′” = 3 and “4′” = 4), where leaves with infection types 0–2 are regarded as resistant and with scores 3–4 as susceptible.

### Marker Analyses

For DNA extraction, a sample of six seeds per inbred line was taken. Genotyping of all lines was done with a custom 10k Infinium iSelect SNP chip that is proprietary to KWS SAAT SE & Co. KGaA, Einbeck, Germany. The SNPs of this assay are partially overlapping with the 5k-SNP assay of Martis et al. (2013) and the 600k-SNP assay of Bauer et al. (2017). About two thousand (2033) SNP markers thereof have been previously mapped by Bauer et al. (2017).

Markers were filtered by population requiring them to have less than five missing values (callrate = 0.06) and three allele states with two calls per state at minimum (minor allele frequency = 0.03). Some markers showed the same segregation across all genotypes from a single population in terms of their allelic state (not necessarily same SNP bases). They were kept for the analysis nonetheless, because segregation can be different between them in other populations. About 700 (P4) to 3000 (P1) markers remained after filtering per population (Supplementary Table S2).

Marker genotypes were converted to 0, 1, 2 coding, where 0 stands for the marker genotype of the susceptible parent, 1 for heterozygotes, and 2 for the marker genotype of the resistant parent. As we expected a single dominant allele from phenotypically segregating plants in heterogenous (also heterozygous) plots (see section “Discussion,” and Supplementary Figure S1) in P2 and P4, the coding for all markers of these populations was adjusted to 0, 1.5, and 2. More precisely, it was a consequence of giving average scores for field plots and doing DNA extraction with bulked seeds. Specifically, plots of genotypes with a dominant marker allele will segregate in a 3:1 (resistant:susceptible) ratio, so that the resulting plot average will be shifted toward the resistant allele (coded as 2). Because of this change in coding, marker effects could be estimated as linear regression coefficients. For the same reasons the allele coding of P1TC was also changed. By crossing each line of P1 with a susceptible tester, only a single allele of the line genotypes can cause resistance. For heterozygous genotypes (H) crossed with a susceptible tester (A) the segregation ratio will be 1:1 (A, H). Consequently, alleles for P1TC were coded 0, 0.5, 1. For better understanding, sketches of expected segregation can be found in the supplement (Supplementary Figure S1).

### Linkage Map

For each population, separate linkage maps were constructed using the R-package ASMap with the mstmap-function (Taylor and Butler, 2017). The ASmap-algorithm creates linkage groups based on a significance threshold. This threshold was chosen by trial-and-error so that linkage groups corresponded to individual chromosomes presented in Bauer et al. (2017) and prevented pseudo-linkage. As a consequence, often more than seven linkage groups were created and had to be merged again. If this was not possible, due to a lack of overlap of markers to public maps or merged groups showing unusual orders or large gaps, markers were discarded. A consensus map was created using the MergeMap online tool (Wu et al., 2008). Chromosome names and marker order were assigned using the overlapping markers from Bauer et al. (2017).

### Phenotypic Data Analysis

Phenotypic means were estimated by using mixed models. The software asreml for R (Gilmour et al., 2009; R Core Team, 2019) was used for all analyses. In a first step, the best rating was selected from three ratings conducted for each plot at the different dates by comparing repeatability. We used the mixed model,

(1) $$y_{i\,j\,k}=\mu +g_{i}+r_{j}+b_{j\,k}+e_{i\,j\,k}$$

where *Y*_*ijk*_ is the response of the *i*th genotype in the *j*th replicate and *k*th incomplete block nested within the *j*th replicate, μ is the intercept, *g*_*i*_ the random effect of the *i*th genotype, *r*_*j*_ the effect of the *j*th replicate, *b*_*jk*_ the effect of the *k*th block nested within the *j*th replicate, *e*_*ijk*_ the error term, repeatability could be calculated by $R\,e\,p=\frac{\sigma_{g}^{2}}{\sigma_{g}^{2}+\bar{v}/2}$, where $\sigma_{g}^{2}$ is the genetic variance and $\bar{v}$ the mean variance of a difference of two BLUEs when fitting genotype as fixed factor (Piepho and Möhring, 2007). As several sets were grown at a single location, the scoring date with the highest repeatability for most sets was chosen and used in the final one-stage analysis. The observations from the location KOS in 2017 were fully discarded because of too low repeatability, even for the trait PH.

To calculate BLUEs for the genotypes, all observations were fit into a single stage analysis with:

$$y_{i\,j\,k\,l\,m\,n\,o}=\mu +g_{i}+y_{j}+l_{k}+{\left(y\,l\right)}_{j\,k}+s_{j\,k\,l}+r_{j\,k\,l\,m}+b_{j\,k\,l\,m\,n}$$

(2) $$+{\left(g\,y\right)}_{i\,j}+{\left(g\,l\right)}_{i\,k}+{\left(g\,y\,l\right)}_{i\,j\,k}+e_{i\,j\,k\,l\,m\,n\,o}$$

Compared to this model (1), the one-stage model was extended by the additional factors year *y*, location *l*, environment (*yl*), set *s* and interaction terms for genotype-year (*gy*), genotype-location (*gl*) and genotype-environment (*gyl*). Indicated by the indices, the factors block (*b*), replicate (*r*), set (*s*), location (*l*) and year (*y*) were nested within each other. For the terms *r*_*jklm*_ and *b*_*jklmn*_ and the error *e*_*ijklmno*_, heterogeneous variance at the level of the environment-wise sets *s*_*jkl*_ was allowed for by using the at() function within the asreml-R. To do a quality check of the model fit (Supplementary Figure S2), residuals were standardized by dividing with the *s*_*jkl*_-specific standard deviation of residuals (=studentized residuals).

To compare variances, genotype was taken as a random factor. Further, different genetic variances for the different sets were expected, so that a set-specific genetic variance was allowed for using the at() function for the genotype effect.

The experiments were laid out in a way that the set was a main plot in the experimental design. This main plot consisted either of the additional lines (B1, B2, B3) or of a mapping population (P1, P2, P2TC, P3, P4). The set was considered as a fixed effect to calculate population-wise genetic variance. Beside the logistical issues, the aim of this experimental set-up was to minimize the errors of a difference within the biparental populations. The least-significant difference (LSD) was calculated by multiplying the average standard error of a difference (average SED) with the 97.5% quantile of the t-distribution with the residual degrees of freedom of the mixed model, that can be extracted from asreml-calculation. The average SED was calculated for each population separately. Before the calculation of the BLUEs, outliers were removed based on the Bonferroni-Holm method with studentized residuals (Bernal-Vasquez et al., 2016). Heritability of the full model was calculated by using an ad-hoc method described by Piepho and Möhring (2007, formula 19), using population-specific variance and error estimates.

### Genotypic Data Analyses

#### QTL Mapping

QTL mapping was performed by a single-point analysis over all markers. To test the significance, the one-stage phenotypic model (2) was extended by a fixed main effect for the *z*th marker (*m*) and a random marker-environment interaction (*myl*). As all populations were developed from separate crosses and different markers were expected to be significant in the different populations, dummy variables were used to account for the stratification. The dummy variable was equal to one when the observation could be referred to a respective population and zero for all other observations. For each population a separate dummy variable *D* was necessary. The model tested with all markers was:

$$y_{i\,j\,k\,l\,m\,n\,o}=\mu +s_{l}+D_{P\,1}\,m_{z\,i}+D_{P\,1\,T\,C}\,m_{z\,i}+D_{P\,2}\,m_{z\,i}+D_{P\,3}\,m_{z\,i}$$

$$+D_{P\,4}\,m_{z\,i}+D_{P\,1}\,{\left(m\,y\,l\right)}_{z\,i\,j\,k}+D_{P\,1\,T\,C}\,{\left(m\,y\,l\right)}_{z\,i\,j\,k}$$

$$+D_{P\,2}\,{\left(m\,y\,l\right)}_{z\,i\,j\,k}+D_{P\,3}\,{\left(m\,y\,l\right)}_{z\,i\,j\,k}+D_{P\,4}\,{\left(m\,y\,l\right)}_{z\,i\,j\,k}$$

(3) $$+g_{i}+d_{j\,k\,l\,m\,n}+{\left(g\,y\,l\right)}_{i\,j\,k}+e_{i\,j\,k\,l\,m\,n\,o}$$

For display reasons the factors of the experimental design of model (2) are summed up in *d*_*jklmn*_ and all interactions regarding the genotype in (*gyl*)_*ijk*_.

The *p*-values could be derived from the Wald-statistics of the fixed effects. The effect sizes for the markers could be estimated from model coefficients. Regression coefficients were interpreted as marker effects. The standard error of marker effects was calculated by taking the square root from the variance of the coefficient estimates.

As P2 and P4 showed similar significant markers, a dummy variable was created that grouped both together so that in an extra run significance and marker effects could be calculated for both populations combined.

We could estimate the explained genetic variance (p_G_) of a fitted SNPs fitted by assessing the associated drop in genetic variance. If the genotype was considered as random factor in model (2), the total genetic variance could be estimated. Every SNP fitted in model (3) should capture some of the genetics so that the amount of genetic variance explained by a SNP could be estimated by comparing genetic variances of model (2) and (3).

Markers were considered as significant when passing a significance threshold. Because of multiple testing the 5%-significance level (α) required adjustments described in the following. Markers being significant in a single-marker model were combined in the order of increasing *p*-values. Only markers that remained significant were considered and are reported.

#### Significance Threshold

We used the method proposed by Piepho (2001), who refers to a method proposed by Davies (1987):

If we consider C as the critical threshold based on a given level of α (=5%) for the Chi-square test statistics of the Wald test, than we can calculate C^∗^ by adding a penalty term to C. It was calculated as follows:

(4) $$C^{*}=C+V\,C^{0.5*k-1}\,\exp\left(-0.5\,C\right)\,2^{-0.5\,k}/\Gamma \,\left(0.5\,k\right),$$

where *V* is the sum of absolute differences between square roots of the likelihood test statistics T of successive marker tests (0 to *n*) along the chromosome:

$$V=\left|\sqrt{T\,\left(\theta_{0}\right)}-\sqrt{T\,\left(\theta_{1}\right)}\right|+\left|\sqrt{T\,\left(\theta_{2}\right)}-\sqrt{T\,\left(\theta_{3}\right)}\right|+\ldots +\left|\sqrt{T\,\left(\theta_{n-1}\right)}-\sqrt{T\,\left(\theta_{n}\right)}\right|$$

As our model was based on single point test, we interpolated the profile of Wald test statistic with the R function smooth.spline (R Core Team, 2019) and extracted turning points θ_*0*_ to θ_*n*_ (sign change of first derivative). An additional turning point with value of zero was added at the start and end of each chromosome.

The global significance level γ of all chromosomes (*z* = 7) was than calculated by:

(5) $$\gamma =z\text{Pr}\left(\chi_{k}^{2}>C+\left(\sum_{i=1}^{z}V_{i}\right)C^{0.5*k-1}\exp\left(-0.5C\right)2^{-0.5\,k}/\Gamma \left(0.5k\right)\right)$$

The variable *k* in formula (4) and (5) depends on the population being studied (Piepho, 2001), in our case *k = 2.*

### Bivariate Model

A bivariate model was used to estimate genetic correlation between P1 and P1TC, between SR and LR and to map QTL for LR.

As LR was scored in 2018 only, the year-interaction terms in model (2) were dropped and the model extended to a bivariate form. To make use of the covariance between the traits it was aimed for unstructured variance for all reasonable fitted factors, but to still allow for the variance heterogeneity of location-set interactions it was possible to reach convergence with unstructured variance for the genotype and residual term (plot error) only. Diagonal variance was fitted for all other factors.

To use a multivariate model for QTL mapping, the model was extended by trait-specific fixed marker effects and random marker-location interaction, the latter again with diagonal variance. All bivariate models were used with marker genotype coding of 0, 1, 2 (A, H, B) for all populations. As covariance was estimated for the factor genotype (CovG), the amount of explained genetic covariance (p_CovG_) was assessed likewise and additionally to p_G_.

### Candidate Genes

No reference sequence of rye is available to directly search for candidate genes. For many contig sequences, gene annotations are available (Bauer et al., 2017). But as the contig sequences do not cover the full rye genome sequence, we compared them with the barley genome. To have a long sequence to compare, we firstly searched the KASP sequence in the Lo7 assembly of the rye database^1^ (Altschul et al., 1997; Bauer et al., 2017). This sequence was then used to search for homologous sequences in the barley database (assembly_WGSMorex^2^; Altschul et al., 1997; Mascher et al., 2017) and corresponding gene annotations.

### KASP Marker Development

Significant markers were transformed into markers for a KASP assay. Primer design for and KASP analysis was performed by KWS SAAT SE & Co. KGaA. Primer design can be found in the Supplement (Supplementary Table S3).

## Results

### Phenotypic Data

#### Stem Rust Resistance

Of the three repeated ratings conducted in each environment most often the final rating had the highest repeatability (0.45–0.99) and was, therefore, used in the one-stage model. The raw data for stem rust in the final model ranged between 0 and 98 % infection. All observations from KOS in 2017 were discarded because of low repeatability for SR (*Rep_*SR3*_* = 0.17–0.84) and PH (*Rep_*PH*_* = 0.50–0.87).

**FIGURE 1:**
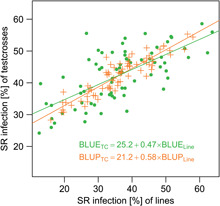
Correlation between lines and respective testcrosses. Best linear unbiased estimators (BLUEs, green) and best linear unbiased predictors (BLUPs, orange) for stem rust infection were calculated based on phenotypic data from six environments and are plotted for the lines (*x*-axis) and respective testcrosses (*y*-axis) of P1. Testcrosses (TC) were regressed on lines (Line) and regression curve and model equation are displayed.

Ranging from resistant to susceptible genotypes (Table 3), the highest genetic variance was observed for P4 followed by P2, P1 and P1TC (Table 4). The reduced genetic variance for P1TC was expected, because the tester allele was always SR susceptible. Indeed, when adjusted means or breeding values of testcrosses were regressed on the performance of the respective lines, a slope of 0.47 and 0.58 was estimated (Figure 1). The genetic correlation between lines and their respective testcrosses was 0.83 (standard error = 0.07).

**TABLE 3:** Statistical parameters for stem rust, leaf rust, plant height, and heading date in four populations (P1–P4), a testcross progeny (P1TC) and an array of breeding lines (B).

	**Value**	**P1**	**P1TC**	**P2**	**P4**	**P1, P2, P4**	**P3**	**B**
Stem rust (%)	Min	7.1	19.7	0.4	4.8	0.4	34.8	6.6
	Max	57.8	54.9	52.4	61.7	61.7	54.9	57.9
	Mean	29.7	37.5	17.4	26.9	24.7	43.4	37.8
	LSD	14.4	14.7	14.6	16.5	17.7	15.0	15.4
	*h*^2^	0.81	0.57	0.84	0.88	0.80	0.00	0.73
Leaf rust (%)	Min	11.0	6.9	0.0	18.5	0.0	5.3	n.a
	Max	46.2	32.5	39.6	46.8	46.8	29.3	n.a
	Mean	19.7	13.7	11.8	25.2	18.9	13.5	n.a
	LSD	11.4	10.9	15.0	11.1	15.9	10.4	n.a
	*h*^2^	0.18	0.03	0.72	0.21	0.13	0.05	n.a
Plant height (cm)	Min	80.0	95.6	96.5	110.3	80.0	84.5	78.3
	Max	117.4	125.6	141.5	137.6	141.5	109.3	114.2
	Mean	98.4	114.7	119.7	123.2	113.8	96.8	93.4
	LSD	7.4	7.1	8.0	8.0	8.2	7.3	7.2
	*h*^2^	0.88	0.82	0.90	0.82	0.87	0.72	0.90
Heading date (day in year)	Min	133.67	131.45	134.06	132.35	132.35	133.00	131.86
	Max	138.48	135.12	138.69	135.74	138.69	139.38	139.59
	Mean	135.45	132.67	136.04	133.88	135.12	136.34	135.17
	LSD	1.65	1.47	1.73	1.75	1.90	1.79	1.70
	*h*^2^	0.72	0.49	0.58	0.50	0.57	0.76	0.81

*Parameters were calculated for biparental populations (P1–P4), the additional breeding lines (B1–B3) and for P1, P2, P4 combined. The minimum (Min), Maximum (Max) and mean were calculated from the best linear unbiased estimators (BLUEs). The least significant difference (LSD) was based on the standard errors of a difference of the BLUEs. Heritability (*h*^2^) was calculated using set-specific genetic variance and average variance of difference. The additional lines have not been assessed (n.a.) for leaf rust.*

**TABLE 4:** Variance components (comp.) with standard errors (SE) for four populations (P1–P4), a testcross progeny (P1TC) and an array of breeding lines (B) for stem rust, leaf rust, plant height, and heading date.

**Factor**		**Stem rust (%)**		**Leaf rust (%)**		**Plant height (cm)**		**Heading date**	
								**(day in year)**	
		**Comp.**	**SE**	**Comp.**	**SE**	**Comp.**	**SE**	**Comp.**	**SE**
Genotype (G)	P1	118.0	23.7	3.6	0.8	51.7	9.9	0.90	0.20
	P1TC	37.4	10.4	0.4	0.2	30.8	6.3	0.25	0.09
	P2	139.6	27.4	74.3	15.8	78.4	14.6	0.56	0.16
	P4	248.0	47.0	4.3	0.9	37.7	7.8	0.41	0.13
	P3	0.0	.	0.7	0.5	17.8	4.2	1.26	0.27
	B1-B3	83.9	32.8	n.a.	n.a.	61.7	20.8	1.86	0.68
Year (Y)		0.0	.	n.a.	n.a.	82.9	126.9	15.64	23.79
Location (L)		122.7	127.1	113.1	110.4	32.0	34.3	14.89	14.19
Y × L		51.1	56.5	n.a.	n.a.	0.0	.	4.30	3.82
Set		138.8	33.8	95.4	43.7	146.4	34.0	1.64	0.44
Replicate^a^		2.8	1.1	19.2	42.9	6.2	15.1	0.66	1.99
Block^a^		12.1	11.1	11.4	10.2	5.9	5.0	0.26	0.27
G × L		3.6	4.1	0.0	.	1.9	0.8	0.14	0.03
G × Y		8.0	3.4	n.a.	n.a.	6.1	1.0	0.27	0.04
G × Y × L		75.9	5.6	n.a.	n.a.	6.6	1.02	0.07	0.04
Residual^a^		67.9	16.8	106.3	16.1	25.4	5.4	1.72	0.35

*Heterogeneous variance was allowed for the different sets so that for each population different genetic variance (G) was estimated. Leaf rust was scored in a single year only so that variance components for the factor year or interactions thereof could not be estimated. Leaf rust was not assessed (n.a.) for the additional breeding lines (B1–B3). ^*a*^Heterogeneous variance was allowed on the level of set nested within year and location. For display the average over all different levels was calculated.*

As the genetic variance enters into the respective heritability calculations as one factor, P1, P2, and P4 had the highest estimates of 0.81, 0.84, and 0.88 (Table 3). The average standard error of a difference (as second factor of heritability) was lowest for P1 and highest for P4 (indicated by LSD, Table 3). The differences in LSD could be explained by the experimental design. The populations shared only a single location for trait assessment (Table 2). Consequently, the LSD was increased when all populations were combined and comparisons were made across populations (Table 3). In addition, the genotype-environment (G × Y × L) interaction was relatively large. Moreover, LSD was increased because populations were generally tested in different trials.

#### Leaf-Segment Test

The LST as seedling test was used to distinguish between APR and ASR. As capacities were limited, only the susceptible parents, the resistant parents or if not available (as in P4) a single resistant progeny as proven in the field test were used. All tested genotypes from P1 were susceptible at seedling stage, whereas the resistance donor from P2 and a resistant genotype from P4 were resistant for all tested isolates (Table 5). Considering median scores, the susceptible parent of P4 showed a single race-specific resistance to isolate 43.1. On a single score basis (data not shown), results for this isolate and isolate 106-5 were heterogeneous for L403: 70% of replicates showed resistant reaction for the former and 12.5% of the latter, the remaining 30 and 87.5% were susceptible, respectively. All other isolates showed uniform resistant or susceptible reactions over all tested genotypes. We concluded APR to be present in P1 and ASR in P2 and P4. However, as not all genotypes from P2 and P4 were tested by LST and mapping was conducted for adult-plant resistance in the field, the seedling resistance could also be caused by another undetected gene.

**TABLE 5:** Median leaf-segment test (LST) scores of selected genotypes.

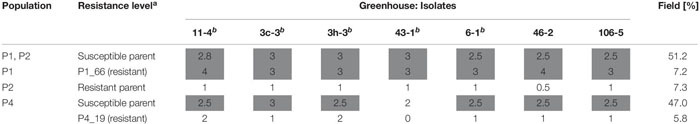

*The susceptible and resistant parents as well as single genotypes (lines) from the respective populations were tested with seven isolates each. For comparison, adjusted means for stem rust scores from the field trials are listed. In LST, scores higher than “2” are considered as susceptible reactions (gray cells). ^*a*^Resistance level was assigned according to information about pretested parents (P2) and according to the field data (P1, P4). ^*b*^Isolates were used in a mixture for field inoculation.*

#### Plant Height and Heading Date

For HD differences of three to 5 days between the earliest and latest genotypes could be observed in each population (Table 3). However, the estimated genetic variance was lower than a single day (Table 4) and thus the trait was neglected. The observed high genetic variance for PH (Table 4) could be explained by the fact that the parents used as donors for SR resistance were derived from non-adapted genetic resources. Both traits, PH and HD, are easy to measure and showed high heritability (Table 3).

#### Leaf-Rust Resistance

**FIGURE 2:**
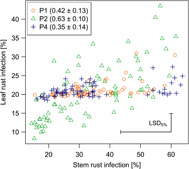
Genetic correlation of stem rust and leaf rust infection. Plotted breeding values (best linear unbiased predictors), estimated correlation with standard errors (brackets) and least significant difference on a 5% level (LSD_5%_) were estimated from a bivariate model allowing for unstructured variance-covariance on the genotype level. Estimations were based on data from 2018 only. Additionally, observations from KOS were removed so that prediction of P4 was based on field data from two and the P1 and P2 from three locations (refer to Table 2).

High genetic variance for LR could only be observed in population P2 (Table 4). In the other populations, variance was negligibly small. A special focus was laid on the correlation between LR and SR, because both traits could be influenced by pleiotropic or closely linked genes. Despite small variation for LR, a significant genetic correlation could be observed in P1, P2, and P4, but whereas in P2 the breeding values ranged from resistant to susceptible, only medium resistant genotypes could be observed in P1 and P4 (Figure 2).

### Genotypic Data

Given the small population sizes and few recombination events (crossing, self-pollination) a high amount of redundant markers was present (Supplementary Table S2). Combined with the size of the linkage maps (Supplementary Table S4) the marker densities were sufficient for the QTL mapping. In all populations, the amount of heterozygous markers was almost equal to the theoretical expectations of heterozygous markers due to the number of self-pollination steps (Supplementary Table S2 and Table 1). P4 had the lowest numbers of polymorphic (filtered) markers (Supplementary Table S2), because an additional backcross step was included in the breeding scheme. The marker distance on the linkage maps was on average between 1.4 cM (P1) and 1.7 cM (P4, Supplementary Table S4). The order of the markers was very similar between the population-specific linkage maps. Differences could only be found on a small-scale level (data not shown).

### QTL Mapping for Stem-Rust Resistance

In P1, we could identify three QTL for stem rust resistance on chromosomes 1R, 2R, and 6R, all having effect sizes between 6.5 and 6.9% points of stem rust reduction (Table 6). Explained genetic variance ranged from 26 (C11974_365) to 31% (C2420_561). Markers from additional peaks of chromosomes 1R and 2R were also significant in single marker fits, but did not remain significant in a combined marker fit and are, therefore, not reported. Combining the three markers in one model, increased the total explained genetic variance to 60%. When only two markers were combined in a model, the best combination was achieved with the markers C2420_561 (1R) and C31257_184 (6R) yielding already 55% of the total explained genetic variance (data not shown).

**TABLE 6:** QTL-mapping results for stem rust (SR) for three populations (P1, P2, P4) seperately and combined (P2+P4) and a testcross progeny (P1TC).

**Pop.**	**QTL/gene**	**Marker**	**Chr.**	**Pos.**	***p*-value**	**Effect**	**SE**	**p_G_**
P1	QTL-SR1	C2420_561	1	121.57	1.50E−04	−6.9	1.8	0.31
		KASP2 (Contig1383)	1	121.57	1.30E−04	−7.0	1.8	0.32
		KASP3 (Contig1648)	1	121.57	1.30E−04	−7.0	1.8	0.32
	QTL-SR2	C11974_365	2	89.06	4.00E−05	−6.6	1.6	0.26
	QTL-SR3	C31257_184	6	236.15	1.10E−04	−6.5	1.7	0.30
	QTL-SR1 + QTL-SR2 + QTL-SR3							0.60
P1TC	QTL-SR3	C31257_184	6	236.15	3.24E−06	−10.5	2.3	0.62
P2	*Pgs1*	C42825_295	7	265.83	2.10E−08	−10.5	2.0	0.58
		isotig25723	7	266.97	8.30E−09	−11.2	2.1	0.64
		isotig12934	7	266.97	8.40E−09	−11.2	2.1	0.64
		KASP1 (isotig12934)	7	266.97	2.10E−09	−12.0	2.0	0.73
P4	*Pgs1*	C42825_295	7	129.31	4.60E−04	−20.6	5.9	0.88
		isotig25723	7	129.31	6.50E−04	−20.4	6.7	0.87
		isotig12934	7	129.31	6.10E−04	−19.8	5.8	0.84
		KASP1 (isotig12934)	7	129.31	3.40E−04	−22.3	6.2	0.97
P2 + P4	*Pgs1*	isotig12934	7	129.31	1.40E−06	−15.1	3.5	.
		KASP1 (isotig12934)	7	129.31	2.00E−06	−17.1	3.6	.

*The populations (Pop) P1, P1TC, P2 and P4 and a combination of P2 and P4 were investigated. The position (Pos.) on the genetic map was based on population specific linkage maps (Chr. = chromosome). The *p*-values were derived from Wald-tests. p_*G*_ gives the explained genetic variance. The effect and standard error thereof (SE) was estimated from model coefficients. When functional KASP markers were available for the respective QTL, results thereof are listed. The SNP chip marker on which they were based on are reported in brackets. For Pgs1, three SNP chip markers are listed because the one with the smallest p-value differs between P2, P4 and P2 + P4 respectively.*

**FIGURE 3:**
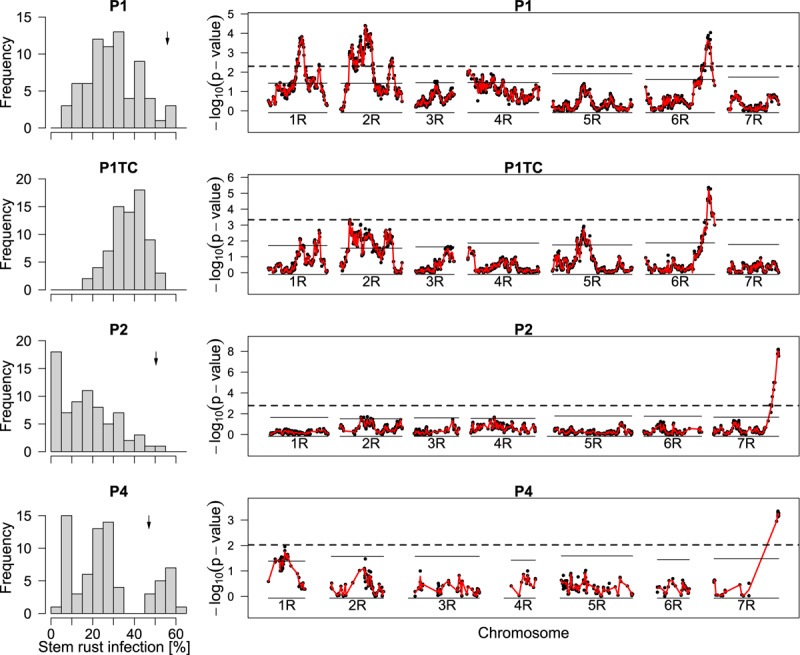
Distribution of phenotypes for stem rust infection and LOD curves from QTL mapping. Histograms display the best linear unbiased estimators (BLUEs) of line populations P1, P2, P4, and P1TC, the respective testcross of P1. The calculation of the plotted BLUEs was based on model (2) without the use of marker data. The BLUEs of the susceptible parents are indicated by a little arrow. *p*-values for single-marker testing along the seven chromosomes 1R to 7R are plotted as –log10 (*p*-value). The *p*-values were derived from model (3). The red line is based on the interpolation of the Wald statistics and was used to calculate the chromosome-wise (solid lines) and global (dashed lines) significant thresholds. Linkage maps were based on the respective populations.

When mapping the testcross population, only a single QTL on chromosome 6R remained significant. For the top marker of chromosome 6R, a single allele change in P1TC had almost doubled effect size (−10.5) compared to a single allele change in P1 (−6.5) so that we concluded dominant gene action according to our marker coding (Figure 3 and Table 6). The QTLs from P1 on chromosomes 1R and 2R were below the threshold in the testcross progenies. Despite this, effects were estimated for the respective markers and the explained total genetic variance increased from 62 to 70% (data not shown).

Selected markers from the SNP array were transferred into KASP assays. Unfortunately, the markers were chosen based on simple fits in QTL mapping software and first year data, so that one of the converted SNPs was located about 15 cM away from C31257_184 and the conversion of a second SNP into a KASP marker resulted in monomorphic response even though the respective SNP segregated. KASP2 and KASP3 were based on markers at the same position as C2420_561 and produced similar results as the respective SNP (Table 6).

**FIGURE 4:**
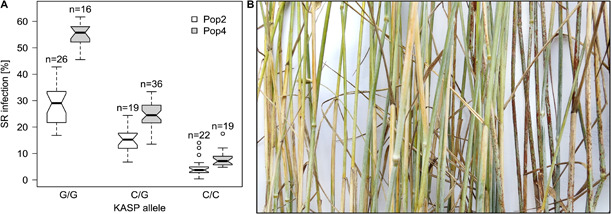
Stem rust infection for genotypes grouped by KASP marker and segregation of heterozygous genotypes in the field. **(A)** Best linear unbiased estimators (BLUES) from genotypes of P2 (white fill) and P4 (gray fill) grouped by the marker alleles of KASP1 on chromosome 7R. Group sizes are given on top of the boxes (*n*). **(B)** Detail of a scored plot. Within a single row, segregation could be observed for genotypes heterozygous for stem-rust scores. The picture was taken in the field. For contrasting purposes, white paper was placed behind the stems.

In P2 and P4, significant markers were located at the distal end of chromosome 7R. Both populations had highly significant markers in common, but still the markers with the lowest *p*-value differed. When both populations were combined, isotig12934, was the most significant marker for both populations. The marker isotig12934 was converted to a KASP assay and yielded even higher significance than the related SNP. The marker effects (codominance) were estimated to cause a reduction of 12.0 and 22.3% points stem rust severity in P2 and P4, respectively (Figure 4A), and to 17.1% points reduction when both populations were combined. As discussed later, the gene was inherited dominantly, so the effects sizes must be doubled. The genetic variance explained by a single marker was with 73 (P2) and 97% (P4) extremely high indicating monogenic inheritance in both populations. We postulate, therefore, a single gene, *Pgs1*, on chromosome 7R in both populations. It could also be possible, that two different genes, one in each population, were segregating at this locus. But even then, the validation of the same marker in two different populations proved the potential for practical breeding.

### QTL Mapping for Leaf Rust Resistance

Given the high amount of genetic variance for LR (Table 4) and genetic correlation with SR (Figure 2), P2 was most informative for QTL mapping. Two QTLs for LR could be identified on chromosome 1R and 7R (Table 7). The marker isotig26262, located on chromosome 1R, had a positive effect of 4.9 on LR indicating that the SR-susceptible parent was the donor. In P1, a significant QTL was located at a similar position as in P2. This could be expected as both populations shared the same susceptible parent (KWL1770_90, Table 1). But the most significant marker from P2 (isotig22192) was monomorphic in P1 and a marker (isotig16666) being at a similar position and polymorphic in P1 and P2, showed opposite effects, so a different gene (QTL-LR1b) was assumed to be present at this locus in P1. The effect in P1 was very small (Table 7).

**TABLE 7:** QTL mapping results for leaf rust (LR) in three populations.

**Pop.**	**Gene/QTL**	**Marker**	**Trait**	**Chr.**	**Pos.**	***p*-value**	**Effect**	**SE**	**p_G_**	**p_CovG_**
P1	QTL-LR1b	isotig26262	LR	1	92.64	1.50E−03	−1.0	0.3	0.17	0.28
		isotig16666	LR	1	82.74	2.50E−03	−0.9	0.3	0.17	0.27
P2	QTL-LR1a	isotig22192	LR	1	82.92	1.90E−04	4.9	1.3	0.24	0.09
		isotig16666	LR	1	82.92	3.60E−04	4.9	1.4	0.24	0.09
	*Pgs1*	isotig12934	LR	7	266.97	4.80E−03	−3.9	1.4	0.11	0.50
			SR			7.60E−14	−15.5	1.9	0.77	
	QTL-LR1a + *Pgs1*		LR						0.34	0.54
P4	QTL-LR2	isotig25476	LR	2	88.93	6.00E−03	−2.0	0.7	0.26	0
	*Pgs1*	isotig12934	LR	7	129.31	1.20E−02	−2.3	0.9	0.20	.
			SR			7.30E−02	−14.0	7.9	0.81	
	QTL-LR2 + *Pgs1*		LR						0.45	.

*In the populations (Pop) P1, P2 and P4, leaf rust QTL were mapped by means of bivariate models. For gene *Pgs1* parameters are reported for stem rust (SR), too. The position (Pos.) on the genetic map was based on population specific linkage maps (Chr. = chromosome). The *p*-values were derived from Wald-tests. p_*G*_ gives the explained genetic variance and p_*CovG*_ the explained genetic covariance. The effect and standard error there of (SE) was estimated from model coefficients. Results for isotig16666 are shown, because isotig22192 was not polymorphic in P1 and isotig26262 not in P2.*

The locus of the tentatively denominated gene *Pgs1* was shown to have also an effect on LR resistance. In P2 the marker isotig12934, which was most significant for SR resistance in both populations (Table 6), was significant for LR resistance in P2 and only slightly below the significance threshold in P4 (Supplementary Figure S3). Looking at results from the only common location DAH for scoring of LR of P2 and P4, isotig12934 could pass the significance threshold (Supplementary Figure S4). For P2, the marker with the smallest *p*-value for LR resistance (isotig28727, data not shown) was located 9 cM away from isotig12934, but the LD was estimated to be 0.93 and correlation of the two markers was significant (*p* < 0.001). Whereas in P2 the QTL LR1a mainly explained genetic variance for LR (24%), a high amount of genetic covariance (50%) was explained by *Pgs1* (Table 7).

Further peaks were identified on chromosome 2R and 4R in P4 (Supplementary Figure S3), though the latter was discarded due to unreasonable allele distributions (number of A alleles <10). The effect sizes for all QTLs in P4 were very small (Table 7).

### Candidate Genes

**FIGURE 5:**
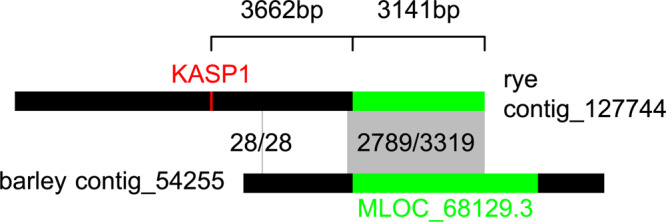
Localization of KASP1 (rye) nearby MLOC_68129.3 (barley) by use of sequence homology. The contig sequences of contig_127744 from the rye Lo7 assembly (top) and of contig_54255 from the barley WGS Morex assembly (bottom) are visualized as horizontal bars. Homologous sequences are connected by gray boxes and number of base identities are given. Position of KASP1 is highlighted in red and position of MLOC_68129.3 in green.

As the KASP1 marker showed high effects in P2 and P4, a BLAST search for potential resistance genes was done. Unfortunately, no full reference sequence of rye is available yet, so that the investigations were based on known gene homology to barley. Using the IPK Rye BLAST server, KASP1 was located on the rye genome contig_127744 (59/61 identities) which is 11,179 basepairs long (Figure 5). When this full sequence was used for a search in the IPK Barley BLAST server its end was overlapping by 3,319 basepairs (84.0% identities) with the contig_54255 from the whole genome assembly of the barley variety “Morex.” On that contig, with 8,587 basepairs length, a NB-LRR disease resistance protein homolog (high confidence gene MLOC_68129.3) was located. Calculating this the other way around, the gene candidate MLOC_68129.3 is directly overlapping 3,187 basepairs with the rye contig_127744 (2,674/3,187 identities). If sequences from the rye contig and MLOC_68129.3 from barley would be merged by the overlapping sequence, the SNP of KASP1 would only be 3,362 basepairs away from the start of MLOC_68129.3, the known barley candidate gene (Figure 5).

## Discussion

For the first time, we mapped three self-fertile rye populations (P1, P2, and P4) for the occurrence of stem-rust resistance genes in the adult-plant stage in the field. Additionally, P1 lines were analyzed as testcross progenies. Because we expected monogenic inheritance, population sizes were rather small (*n* = 68–70). Most probably, we found a single resistance gene segregating that caused most of the genetic variance in P2 and P4 and was also active in seedling stage. In P1, however, a clear quantitative inheritance acting only in APR with at least three QTL was found of which one was inherited dominantly as revealed by the TC progenies.

To the best of our knowledge only Tan et al. (1976, 1977) and Solodukhina and Kobylyansky (2007) proposed names for stem rust resistance genes in rye. With note on the tentativeness of their names, research groups used different systems (alphabetical and numerical) for gene denomination. To avoid confusion with stem rust resistance denomination in the other small-grain cereals, we propose the name *Pgs1* (*Puccinia graminis* f. sp. *secalis*) for the identified resistance gene. We are aware that gene denominations require Mendelian segregation ratios and thus consider the name as temporary. However, we could clearly separate resistant and susceptible plants in the field (Figure 4) and the proportion of explained genetic variance amounted to 73% in P2 and 97% in P4 (Table 6). Thus, we could assign this high variance to a single resistance locus. Further, our results do not rule out that in P2 and P4 two different genes are segregating at the same locus. Both populations, however, were based on genetically different genetic resources. The other significant QTL are referred to with a “QTL-SR” and “QTL-LR” denomination.

### Consequences of Mixed-Model Calculation

The mixed-model framework was a powerful tool to solve analytical challenges we were confronted with. The two main issues were (1) an unbalanced experimental design with only single overlapping environments between the mapping populations combined with heterogeneous error structure and (2) the combination of DNA analysis from bulked seeds and averaged plot scores for heterozygous genotypes. Further, the extension of the mixed model to a bivariate form allowed us not only to estimate genetic correlation between testcross and line performance and between SR and LR infection, but also to use estimated covariance between SR and LR to map the latter by an improved model. The model improvement from a single to a multivariate model could be shown by comparisons of AIC and BIC. If a bivariate model (LR and SR) for phenotypic data was fitted with zero covariance (diag-option) the AIC was larger than with estimated covariance (us-option) for the factors genotype and residual. The more conservative measure BIC behaved the opposite way; it should be kept in mind, however, that the penalty term in BIC is not well defined and the definition used in mixed model package tends to favor too simple models (Pauler, 1998). If bivariate models were reduced to populations with high correlation between traits (P2) also the differences in AIC increased.

Besides the great flexibility of mixed models, we were also confronted with some challenges. Firstly, the use of mixed models increased the computation time compared to simple linear marker regression with averaged genotypic means. To calculate significance for approximately 5000 markers and more than 4800 phenotypic observations took 4–5 h computation time on a standard PC (Intel^®^ Core^TM^ i5-6600K processor) instead of 5 min for a simple regression approach with BLUEs. Consequently, well-known, but computational intensive procedures like significance threshold calculation by permutation (Churchill and Doerge, 1994) or confidence interval (CI) estimation by bootstrapping (Visscher et al., 1996) could not be applied. However, alternatives to the mentioned methods proved to be well suited too. The significance threshold calculated by Piepho (2001) proved to give reasonable results and confidence intervals are mainly defined by effect (explained variance) and population size (Darvasi and Soller, 1997). Following the tables published by Darvasi and Soller (1997), with a given standardized gene effect of 2 (maximum), that is not unrealistic at least for P2 and P4, and a population size of 200 the 95%-CI would still be 4.1 cM large. Beside the side effect that only markers being polymorphic in both populations were kept in the model, the combination of P2 and P4 increased the population size and thus should have reduced the confidence interval. If the top marker isotig12934 was considered as center of the CI, two or three more markers could be found within, using the linkage maps from P2 and P4, respectively. Indeed, the markers isotig25723 and isotig12934 were mapped at the exact same positions in P2 and P4 and thus must both be tightly linked to *Pgs1*. Secondly, the percentage of phenotypic variance (R^2^) that is commonly combined with the heritability measure to assess the explained genetic variance (p_G_) is not simple to extract from mixed model calculations. Even though procedures for calculating *R*^2^ for mixed models have been described (Piepho, 2019), we are mainly interested in the amount of explained genetic variance estimated in the pure phenotypic model. Thus, we could subtract the genetic variance from a model with respective (fixed) marker effects from the genetic variance of the pure phenotypic model to estimate p_G_. Malosetti et al. (2008) used a similar approach but compared diagonals of G matrices directly. Problematically from a statistical point of view was that for the estimation of random effects, normality is a prerequisite. If only single genes cause genetic variation, like in P2 or P4, the estimation of genetic variance by modeling the genotype as polygenic random effect in model (2) is not ideal. Additionally, the extension of the phenotypic model (model 2) by fitted markers (model 3) could also lead to reduction in other variance parameters. This explains the fact that even though the genetic variance for LR in P1 and P4 was almost zero (Table 4), the genetic correlation for LR and SR was significant and QTL mapping still showed significant QTL. However, the effect sizes of these QTLs were very small (Table 7).

### Challenges Using Self-Incompatible Rye Populations as Resistance Donors

The (initial) donors of resistance in P1, P2, and P4 were genetic resources from Russia, i.e., self-incompatible populations. They were crossed with a self-fertile elite line, thus allowing self-pollination in the following generations because our self-fertility is dominantly inherited. Inbreeding by strict self-pollination provided a challenge due to strong inbreeding depression reducing the number of progenies from one self-pollination step to the next. Doubled-haploid techniques are not feasible in rye. For shortening the procedure, we used unselected F_2_ families and a backcross family subsequently self-pollinated to F_3_ or F_4_ generation. Consequently 50 (P4), 25 (P2) and 12.5% of heterozygous genotypes were expected and observed on the marker level (Supplementary Table S2). If both, the DNA and the phenotype would had been investigated on single plant level, no further consideration concerning marker coding and inheritance mode had to be done. However, our approach of investigating whole plots with several plants combined with bulked DNA analysis, required special attention in the analysis and interpretation of results. A heterozygous marker call was derived from a plant (seed) mixture of A, H and B genotypes at a 1:2:1 ratio (Supplementary Figure S1) and this ratio must be observed in every field plot of a heterozygous genotype, too. As average scores for whole plots were given in our study it would also result in intermediate scores between the two (resistant and susceptible) parental types. In rows of heterozygous (heterogeneous) genotypes of P2 and P4 it was possible to clearly separate between resistant and susceptible plants (Figure 4B). A ratio of 3:1 (resistant:susceptible) plants was observed, indicating a dominant inheritance by a single gene. Consequently, plot averages were expected to be 50% better than the intermediate level. To account for this, the marker coding was adjusted to 0, 1.5, and 2 instead of 0, 1, and 2 for A, H, and B genotypes.

The great advantage of this scheme compared to single-plant investigations was that it was possible to replicate genotypes within and at different locations and to get reliable estimates of stem rust infection in the adult plant stage. Nevertheless, the assessments of heterogeneous plots were a challenge and they tend to have larger errors and artificially influence the genotype-environment interaction if by chance the ratios do not fully meet the expectations. Whereas in P2 and P4 the segregation was evident in the field, it was less clear in P1, so that marker coding remained unchanged. By use of testcrosses, dominant gene action of QTL-SR3 could be shown. Besides the deduced dominance by P1TC, the LOD curve (Figure 3) also showed that this was a major gene in the population. Still, only 62% of genetic variance was explained by it (Table 6), so that further genes may be present. The estimated slope of the regression between line and testcross performance (Figure 1) alone did not imply dominant action. For homozygous lines, a single dominant gene would lead to a slope of one. Given a high inbreeding generation in P1 (Table 1) it is most probable, that the other QTLs in P1 had recessive or codominant gene action adding to QTL-SR3. In this case, statistical models with cofactors (composite interval mapping; Jansen and Stam, 1994) could improve the mapping procedure.

### Co-localization of a Stem Rust and Leaf Rust Resistance Gene

We could identify the stem rust resistance gene *Pgs1* to be identical (pleiotropic) or at least closely linked to a leaf rust resistance gene (gene complex). As mentioned previously for SR, this was also not confirmed by Mendelian segregation ratios. Still, the markers at this locus were significant for both traits and explained a high amount of genetic covariance p_CovG_ (Table 7). To decide whether this holds true for both, P2 and P4, the correction of the critical significance threshold was important. Whereas in P2 a highly significant peak could be found at the position of *Pgs1* (Table 7 and Supplementary Figure S4), in P4 also a peak was present, but less clear. The *p*-value for the marker isotig12934 remains just slightly below the adjusted threshold and peaks on chromosome 2R and 4R had smaller *p*-values. However, when mapping was done with data from DAH, the only common location of P2 and P4 (Table 2), the profiles of Manhattan plot for P4 looked a bit different compared to data from all locations combined (Supplementary Figure S3) and markers linked with *Pgs1* were slightly passing the threshold also in P4 (Supplementary Figure S4). Given that LR races are extremely diverse (Wilde et al., 2006), the race (and virulence) composition could differ between the locations. When reducing the data for the bivariate model to a single year observation, in P4 also the *p*-value for isotig12934 and SR did not pass the significance threshold anymore (Table 7). We explain this by data from a single location (KOS) that was not well correlated with data from the other locations. But proving that the data from three locations can give valid results, the LR-QTLs identified in this study have already been reported by other authors (Wehling et al., 2003; Roux et al., 2004).

Furthermore, it is highly plausible that the LR resistance linked to *Pgs1* has already been described by Wehling et al. (2003). They identified a leaf rust of caused by (*P. recondita*) resistance gene (*Pr2*) in rye at the distal end chromosome 7R. When the sequence of the reverse primer of cMWG682 linked to *Pr2* was searched for in the IPK rye contig database^3^ (Altschul et al., 1997; Bauer et al., 2017) it fully matched with contig_1359301, a contig that has already been mapped by Bauer et al. (2017) at 138.7 cM. It is the same position as if *Pgs1* would be projected on the map from Bauer et al. (2017). The marker cMWG682 has also previously been used to trace the *Lr37/Yr17/Sr38* cluster in wheat (Seah et al., 2001; Helguera et al., 2003; Li et al., 2016), and *Sr38* is reported to be introgressed from *Aegilops ventricosa* (Seah et al., 2001).

Assuming that in P2 and P4 the same gene (complex) was causing resistance for SR and LR resistance, differences in effect size and explained genetic variance (Table 7) could also be explained by differing genetic background mainly passed over by the susceptible parents. In P2, also the interaction with the second LR QTL on chromosome 1R could have played a role. This gene was inherited from the susceptible parent (KWL1770_90) and thus should also be found in P1. However, missing segregation for this QTL in this population impeded any significance, but instead we identified a significant QTL with opposite effect at the same position (different marker, Table 7). Roux et al. (2004) also identified three LR resistance genes (*Pr3*, *Pr4*, *Pr5*) from different resistance donors at similar positions on chromosome 1R. Assuming that the susceptible line of P1 and P2 is carrier of a LR resistance (QTL-LR1a), that was segregating in P2 and was fixed for resistance in P1, there must have been an additional gene segregating in P1 (QTL-LR1b) coming from the stem rust resistance donor and thus having an opposite effect. It was not possible to properly separate those two QTL because we observed marker discrepancies in this region of the linkage maps (Supplementary Figure S5). Interestingly, in Roux et al. (2004) the order of their markers was also inconsistent in the same genomic region. New populations constructed from single plants of the respective populations could give more evidence for clarification of this phenomenon.

### Search for Candidate Genes

Given the clear significance and high effect for markers linked with *Pgs1*, we tried to find candidate genes. Already Bauer et al. (2017) published potential candidate genes in their supplement. We could not find a potential resistance gene candidate there and searched in the barley genome. Indeed, a NB-LRR disease resistance protein homolog was detected. Based on merged sequences of barley and rye it was only 3360 bp away from our functional KASP marker. As not the full resistance gene was located on the respective rye contig (Figure 5), it could not be detected by Bauer et al. (2017). In many plant species, resistance genes often encode for the family of intracellular nucleotide-binding leucine-reach repeat receptors (NB-LRR, Dangl et al., 2013), e.g., *Sr50* in wheat (Mago et al., 2015).

Nevertheless, it must be stressed that only a continuous genome sequence of rye can give us exact estimates of physical distances between markers and maybe other resistance genes. However, studies on the sequence homology of rye with other grass species (Huang et al., 2002; Martis et al., 2013) showed that large chromosomal regions are highly similar. The distal end of chromosome 7R is homologous to the top of chromosome 2H in barley and to the chromosomes 2A, 2B, and 2D in wheat. The compared sequence motifs remained in the same order (Martis et al., 2013). Further, the sequence changes by mutation were estimated to be very low (2 to 5 changes over 1 million years in an intron sequence) and most likely barley and rye diverged from a common ancestor 11 million years ago (Huang et al., 2002). The probability that there is a gene homolog of MLOC_68129.3 in rye on a homologous segment is high, but sequences of the barley and rye contig could not be aligned for the full length. There was a gap of 2502 basepairs at the start of the barley contig (Figure 5). This showed that sequence changes must have taken place in the past. As any amount of DNA could have been inserted or deleted after the segregation of rye and barley from a common ancestor, it cannot be estimated how big the difference between KASP1 in rye and MLOC_68129.3 in barley really is.

### Potential Use of the New Resistance Genes

Definitions of APR and ASR are often discussed in the context of durability (Ellis et al., 2014; Mundt, 2018). However, durability cannot simply be attributed to one of those classes. Flor (1971) introduced the gene-for-gene concept as arms race between the virulence of the pathogen and avirulence of the host. Following the idea of an arms race, specific ASR genes can simply be overcome by the emergence and spread of new races, so that APR like QTL-SR3 identified in P1 seems to be promising for more durable resistance. In the rye stem rust pathosystem, we can find a high diversity of races (Miedaner et al., 2016). At JKI Kleinmachnow, isolates of naturally occurring stem rust in Germany and Poland were analyzed by producing single-spore isolates and testing them on 15 differential lines (data not shown). Out of 196 isolates analyzed from 2013 until 2018, 136 isolates showed a different pattern regarding resistant and susceptible reactions on the differential lines. One of those lines (D26) was carrier of *Pgs1* (parent of P2). Even though it could not be infected by any of the collected 196 isolates, we cannot make any reliable statements about the race-specificity or even durability of that gene. In the recent past, there was no selection pressure by SR-resistant cultivars in Germany that would have boosted the propagation of more virulent races. Important for the emergence of new races is the sexual recombination on the barberry plant (*Berberis vulgaris*) where several virulences can be combined resulting in new and more virulent races. Systematic eradication of the barberry in the United States (Peterson, 2018) and Europe (Bary, 1865) aimed to break the cycle of the pathogen, but today it is assumed that the pathogen (at least in the United States) can persist in the uredinial stage by traveling from south to north using volunteer plants along roadsides as bridge (Leonard and Szabo, 2005). In that context, somatic exchange of genes, shown by Chen et al. (2017) and postulated for the emergence of the Ug99 lineage of wheat stem rust (Li et al., 2019) may play a major role for pathogen diversity. Further, spores can be transported over long distances (Hirst et al., 1967) making the control of the pathogen even more difficult. In Germany, rye stem rust could be isolated from collected barberry leaves (Miedaner et al., 2016), showing that barberry is still important in the life cycle of stem rust in Germany.

Combining two or more resistance genes in a variety, known as pyramiding or stacking, should increase the barrier for the pathogen to be overcome. Concerning the degree of infection, not all combinations are promising. For example in wheat, *Sr24* and *Sr26* combined together give a similar resistance reaction compared to each gene alone (Ellis et al., 2014). Further, if pyramids are developed from already released single R-gene carrying varieties, they can again be overcome by single-step mutations (Ayliffe et al., 2008). As example for the other side, *Sr2* in wheat increases resistance of other genes and can be helpful to identify more minor genes. Those combinations are often referred to as *Sr2*-complex (Singh et al., 2014).

Quantitative inheritance as we concluded for P1 is rather uncommon in cereal rust pathosystems. One reason therefore may be that many resistance genes were mainly characterized by seedling tests (McIntosh et al., 1995) and the minor genes causing quantitative distributions are active in the adult-plant stage only. It could also be possible, that P1 shows a quantitative distribution caused by an intermixture of reactions of several isolate-specific R-genes, but all isolates used as mixture for field inoculations plus additional isolates (Supplementary Table S1) were tested separately for selected genotypes from P1 by LST and did not show any resistant reactions. The fact that we could identify quantitative resistance in one out of three rye populations may be an indicator that this type of resistance is more common in rye than it was thought to be, and it may be worth also in the wheat pathosystem to focus on both, seedling- and adult-stage testing.

It was surprising that we could find the same R-gene in two independently developed populations. A reason may be that both resistance donors were derived from Russian breeding material (cross-pollinating populations), although from different resources (Table 1). Solodukhina and Kobylyansky (2000, 2007) also found resistances in Russian material. In comparison, Tan et al. (1976, 1977) proposed six resistance genes for the forma specialis *secalis* and eight resistance genes for forma specialis *tritici*, by using three different populations only, all from the United States.

Summing all up, the stem rust resistance genes *Pgs1* and QTL-SR3 are valuable sources for breeding hybrid rye. Firstly, they act dominantly so that the introgression in only a single gene pool is necessary. Secondly, potential differences in resistance type (APR vs. ASR) indicate different resistance mechanisms what can be promising for successful gene pyramiding and thirdly, if a gene complex of *Pgs1* with *Pr2* (or a different LR resistance) can be proven in further studies, it can be appealing as it allows to breed for SR and LR resistance in a single step.

Research on resistance mechanisms conferred by different genes will help to explain durability, at least partially. Until then, every resistance gene is a valuable resource and good markers will help breeders to include them in their breeding material, preferably stacked.

## Data Availability Statement

The datasets for this manuscript are not publicly available because the breeding material investigated (populations P1, P1TC, P2) and marker sequences from the SNP chip are property of KWS LOCHOW GmbH, Ferdinand-von-Lochow-Str. 5, 29296 Bergen, Germany and can only be shared by personal request directed to Dr. Andres Gordillo. Breeding material developed at the University of Hohenheim (population P4) can be requested from the corresponding author.

## Author Contributions

TM and BS designed the experiments. A-KS and KF produced inoculum for field experiments and conducted the leaf-segment tests. BS and SK developed the breeding material for QTL mapping. BS, F-JF, and KM provided the additional breeding lines. All authors conducted the field trials except MS and VK who were responsible for the DNA extraction, analysis with the SNP chip and KASP design and analysis. PG analyzed the data and wrote the manuscript. H-PP supervised the statistical issues. TM edited the manuscript. All authors read and approved the manuscript.

## Conflict of Interest

BS, JE, AG, and MS were employed by the company KWS LOCHOW GmbH. VK was employed by the company KWS SAAT SE & Co. KGaA. FF and DS were employed by the company HYBRO Saatzucht GmbH & Co. KG. KM, RK, and MN were employed by the company Danko Hodowla Roślin Sp. z o.o.

The remaining authors declare that the research was conducted in the absence of any commercial or financial relationships that could be construed as a potential conflict of interest.

## Footnotes

1 https://webblast.ipk-gatersleben.de/ryeselect/

2 https://webblast.ipk-gatersleben.de/barley_ibsc/

3 https://webblast.ipk-gatersleben.de/ryeselect/

## Abbreviations

APR: adult-plant resistance
ASR: all-stage resistance
BLUE: best linear unbiased estimator
BLUP: best linear unbiased predictor
CI: confidence interval
HD: heading date
KASP: competitive allele specific polymerase chain reaction
LD: linkage disequilibrium
LR: leaf rust
LSD: least significant difference
LST: leaf-segment test
NB-LRR: nucleotide binding leucine reach repeat
PH: plant height
QTL: quantitative trait loci
SNP: single nucleotide polymorphism
SR: stem rust.
