# Supplementary material for: Mapping Stem Rust (Puccinia graminis f. sp. secalis) Resistance in Self-Fertile Winter Rye Populations
Source: Front Plant Sci. 2020 May 26;11:667. doi: 10.3389/fpls.2020.00667 (PMC7265987; doi:10.3389/fpls.2020.00667)
Supplement: Supplementary file 1 [file Data_Sheet_1.docx]

## Supplementary material

### Supplementary tables

**Table S1 Virulence reaction of single pustule isolates (rows) on 15 rye differential lines (columns).**

|  |  | **Differential lines** | | | | | | | | | | | | | | |
| --- | --- | --- | --- | --- | --- | --- | --- | --- | --- | --- | --- | --- | --- | --- | --- | --- |
|  |  | **D26** | **D31** | **D36** | **D30** | **D48** | **D25** | **D35** | **D47** | **D44** | **D37** | **D45** | **D41** | **D33** | **D46** | **D43** |
| **Isolates** | **6-1**^a^ | 0 | 0 | 0 | 0 | 4 | 0 | 0 | 2 | 3 | 3 | 3 | 1 | 3 | 3 | 3 |
|  | **3c-3**^a^ | 0 | 0 | 0 | 0 | 4 | 0 | 0 | 3 | 3 | 2 | 3 | 3 | 3 | 3 | 3 |
|  | **106-5** | 0 | 0 | 0 | 2.5 | 2.5 | 3 | 0 | 2 | 2 | 2 | 3 | 3 | 3 | 3 | 3 |
|  | **3h-3**^a^ | 0 | 2 | 0 | 3 | 0 | 3 | 2.5 | 2 | 2 | 3 | 2 | 4 | 4 | 3 | 3 |
|  | **11-4**^a^ | 0 | 0 | 2 | 0 | 0 | 0 | 3 | 3 | 3 | 3 | 3 | 3 | 3 | 3 | 4 |
|  | **46-2** | 0 | 0 | 0 | 0 | 0 | 0 | 3 | 2.5 | 4 | 3 | 3 | 4 | 4 | 3 | 4 |
|  | **43-1**^a^ | 0 | 0 | 0 | 0 | 1 | 3 | 4 | 2.5 | 2.5 | 4 | 3 | 4 | 4 | 3 | 2.5 |

The scores 0, 1 and 2 were given if no or only minor infections were present and line is regarded as resistant (no fill). If the score was 2.5, 3 or 4 differential lines were regarded as susceptible to the certain isolate (grey fill). ^a^Isolates were used for field inoculation.

**Table S2 Characteristics of genetic markers used for QTL-mapping**.

| **Pop** | **N** | **Redundant** | **A** | **B** | **H** | **Missing** |
| --- | --- | --- | --- | --- | --- | --- |
| all | 4676 | 204 | 0.70 | 0.18 | 0.10 | 0.02 |
| P1 | 2951 | 1022 | 0.50 | 0.40 | 0.08 | 0.02 |
| P2 | 1473 | 276 | 0.38 | 0.34 | 0.25 | 0.04 |
| P4 | 696 | 178 | 0.23 | 0.25 | 0.45 | 0.07 |

Marker were coded in the parental alleles A (susceptible), B (resistant) and H (heterozygous) and were filtered population-wise by having four or less missing values (Call rate = 0.06) and three allele states with 2 calls per state (A,H,B) at minimum (Minor allele frequency = 0.03). The number of markers (N) and the number being redundant is reported as well as the fraction of the alleles and missing values.

**Table S3 Marker sequences for KASP design.**

| **Name** | **Left** | **SNP** | **Right** | **SNP name** |
| --- | --- | --- | --- | --- |
| KASP1 | ACATACTTGTTTACAAGTATACTCCTCAGA | S | CTAKCTAGGCCGCAACACAAACCGTATCGC | isotig12934 |
| KASP2 | AACATCAGCAACATACTGACAGGGAAATGC | Y | TGGCTAAAATAAAGGCTGGCAATTTCAATG | Contig1383 |
| KASP3 | CAAAATAAATCAAAATCAGCACAACCCACA | R | TAACGAAGATGTGACACACAATGAGAGAAG | Contig1648 |

Flanking sequences (left and right) of the candidate markers (SNP name) from the 10k Infinium iSelect chip were used to construct primers for competitive allele specific polymerase chain reaction (KASP) genotyping assay. Sequence and single nucleotide polymorphism (SNP) is reported in FASTA format.

**Table S4 Comparison of constructed linkage maps**.

| **Chr** | **MergeMap** | | **P1** | | **P2** | | **P4** | |
| --- | --- | --- | --- | --- | --- | --- | --- | --- |
|  | **cM** | **n** | **cM** | **n** | **cM** | **n** | **cM** | **n** |
| **1** | 464.6 | 355 | 214.7 | 152 | 245.9 | 165 | 73.8 | 49 |
| **2** | 465.6 | 349 | 223.3 | 179 | 261.4 | 173 | 109 | 79 |
| **3** | 441.4 | 263 | 140 | 109 | 184.2 | 109 | 143.6 | 73 |
| **4** | 436.3 | 359 | 258.5 | 172 | 265.3 | 155 | 63.5 | 40 |
| **5** | 533.1 | 411 | 290.1 | 212 | 349.5 | 222 | 151.7 | 108 |
| **6** | 328.3 | 256 | 251.3 | 152 | 238.5 | 159 | 64.5 | 38 |
| **7** | 462.6 | 323 | 183.6 | 144 | 281.6 | 158 | 139 | 51 |
| **all** | 3131.9 | 2316 | 1561.5 | 1120 | 1826.4 | 1141 | 745.1 | 438 |
| **Avg cM** | 1.35 | | 1.39 | | 1.60 | | 1.70 | |

Using ASmap (Taylor and Butler 2017) linkage maps were calculated for all individual populations (P1 to P4) and a consensus map (MergeMap, Wu et al. 2008) combined all markers. The distance (cM) and number of markers (n) was based on maps where duplicated marker positions were removed. The average marker density (Avg cM) is the full map length divided by the number of markers.

### Supplementary figures


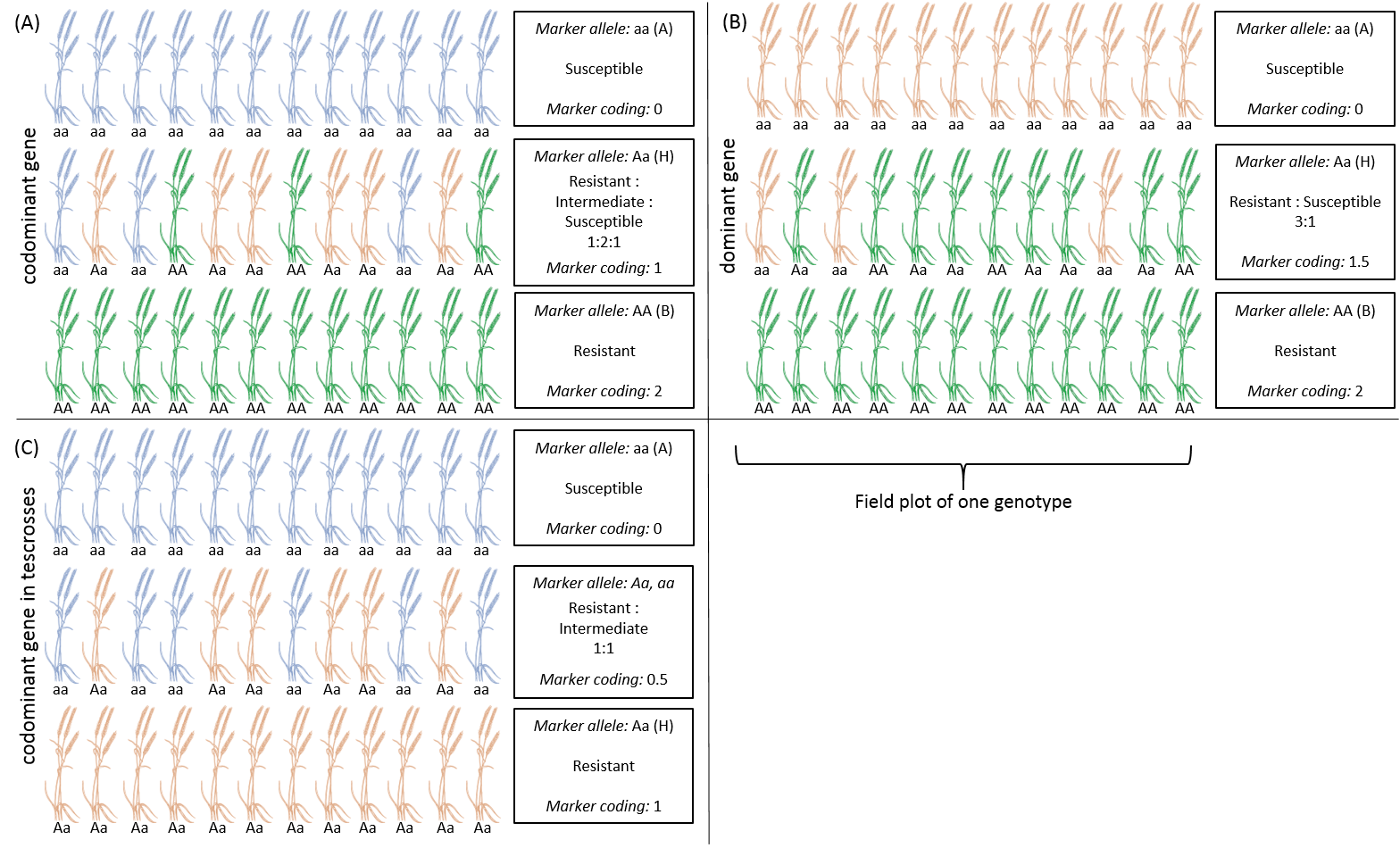


**Figure S1 Visualisation of phenotypic (blue=homozygous susceptible, green=homozygous resistant, orange=heterozygous) and genetic (letters) segregation of the three possible allelic states observed for a single plot**. Each row represents single plants of a genotype. The DNA was extracted from a bulked seed sample and markers cluster into parental allele A and B and heterozygous (H) allele (Marker allele). The heterozygous and heterogeneous genotypes (middle rows) were not consisting of uniformly heterozygous plants. Instead, they were segregating into different allele states (visualized by letters). If the marker alleles (letters) are corresponding to a respective phenotype (color) and there is codominant gene action **(A)**, expected average scores for a field entry of heterozygous genotypes (on DNA extraction level) are also intermediate between the two parental types. Consequently markers are coded 0, 1, 2 (A, H, B). If dominant gene action can be expected based on segregation ratios in the field plots **(B)**, average scores for the heterozygous field entry are expected to be 50% better (more resistant plants). Thus, marker coding was changed to 0, 1.5, 2. In the case of observed testcrosses with a susceptible tester **(C)**, an intermediate state between the heterozygous and susceptible stage is present. Note, that the marker allele coding by small (a) and capital (A) letters does not imply recessive and dominant gene action.


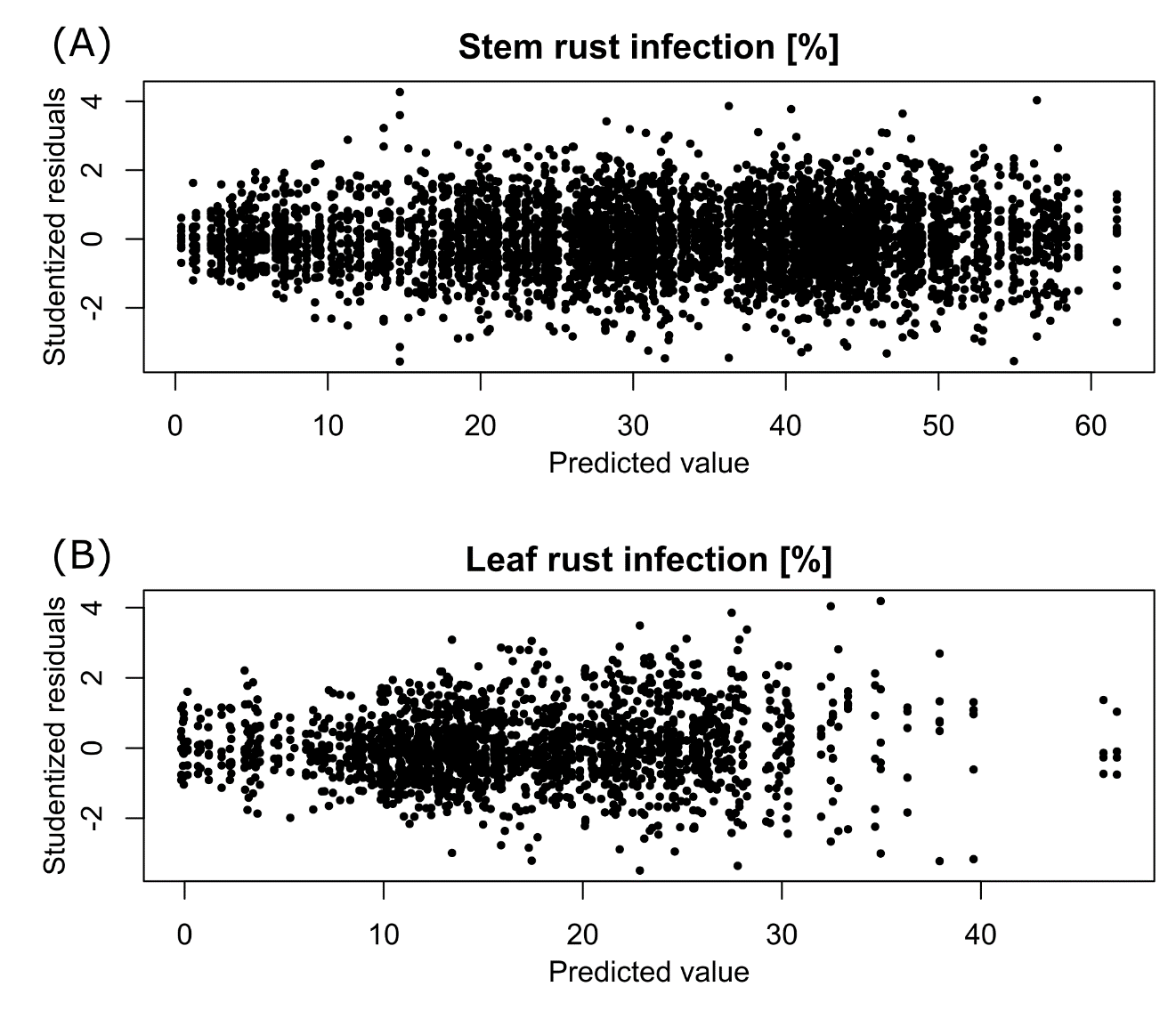


**Figure S2 Studentized residuals plotted vs. the predictions from the phenotypic one-stage model**. All observations for all tested materials, except the observations from the location KOS in 2017, were combined in the model. Outliers were removed using the Bonferroni-Holm method with studentized residuals according to Bernal-Vasquez et al. (2016). Residual plots are shown for stem rust **(A)** and leaf rust **(B)** as both traits were used for QTL mapping.


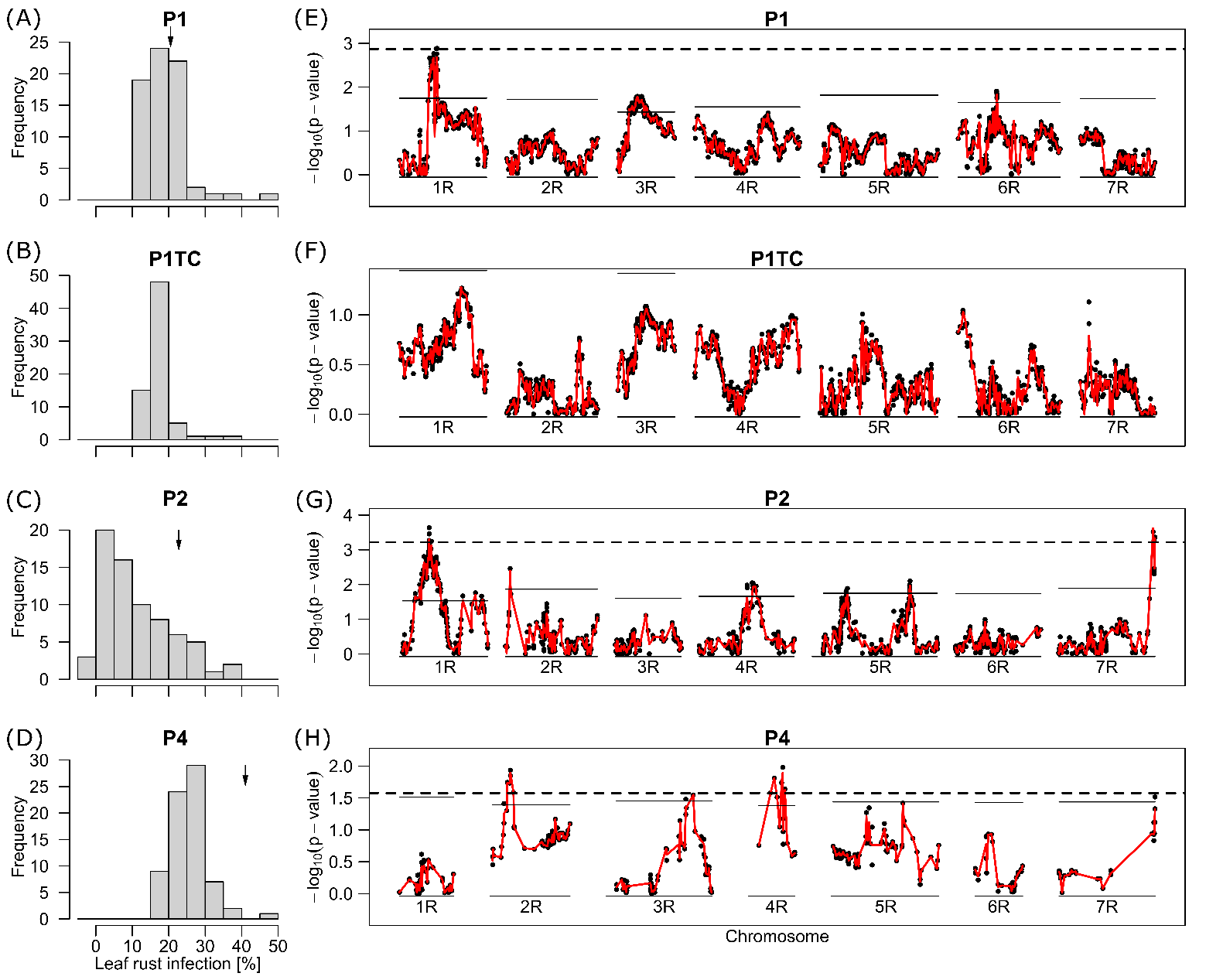


**Figure S3** **Distribution of phenotypes for leaf rust infection and LOD curves from QTL mapping**. **(A-D)** Histograms display the best linear unbiased estimators (BLUEs) of line populations P1, P2, P4 and P1TC, the respective testcross of P1. The calculation of the plotted BLUEs was based on model (2) without the use of marker data. The BLUEs of the susceptible parents are indicated by a little arrow. **(E-H)** p-values for single-marker testing along the seven chromosomes 1R to 7R are plotted as -log10 (p-value). The p-values were derived from model (3). The red line is based on the interpolation of the Wald statistics and was used to calculate the chromosome-wise (solid lines) and global (dashed lines) significant thresholds. Linkage maps were based on the respective populations.


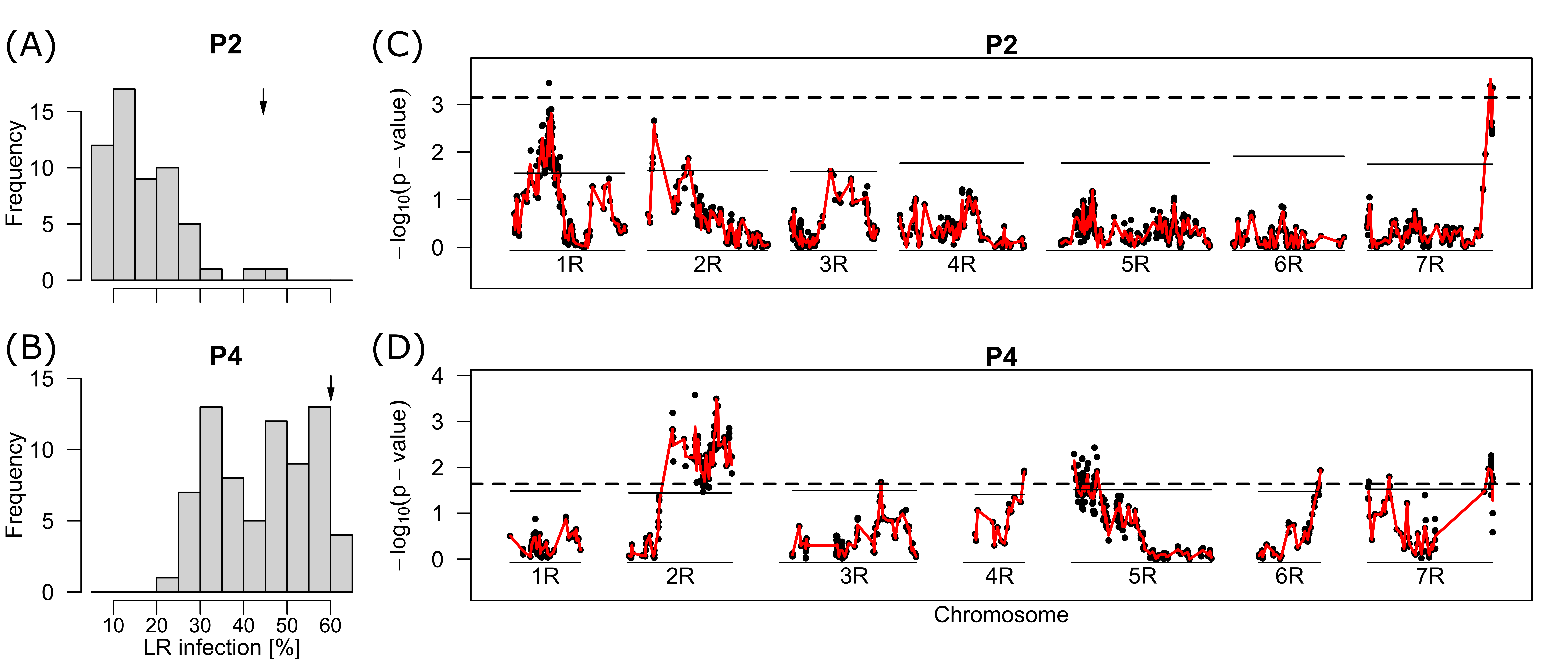


**Figure S4 QTL mapping results for population P2 (A) and P4 (B) and leaf rust resistance with data from the common location DAH in 2018**. Both populations were commonly grown and rated only in this location. **(A,B)** Histograms display the best linear unbiased estimators (BLUEs). The calculation of the plotted BLUEs was based on model (2) without the use of marker data. The BLUEs of the susceptible parents are indicated by a little arrow. **(C,D)** P-values for single-marker testing along the seven chromosomes 1R to 7R are plotted as -log10 (p-value). The p-values were derived from model (3). The red line is based on the interpolation of the Wald statistics and was used to calculate the chromosome-wise (solid lines) and global (dashed lines) significant thresholds. Linkage maps were based on the respective populations.


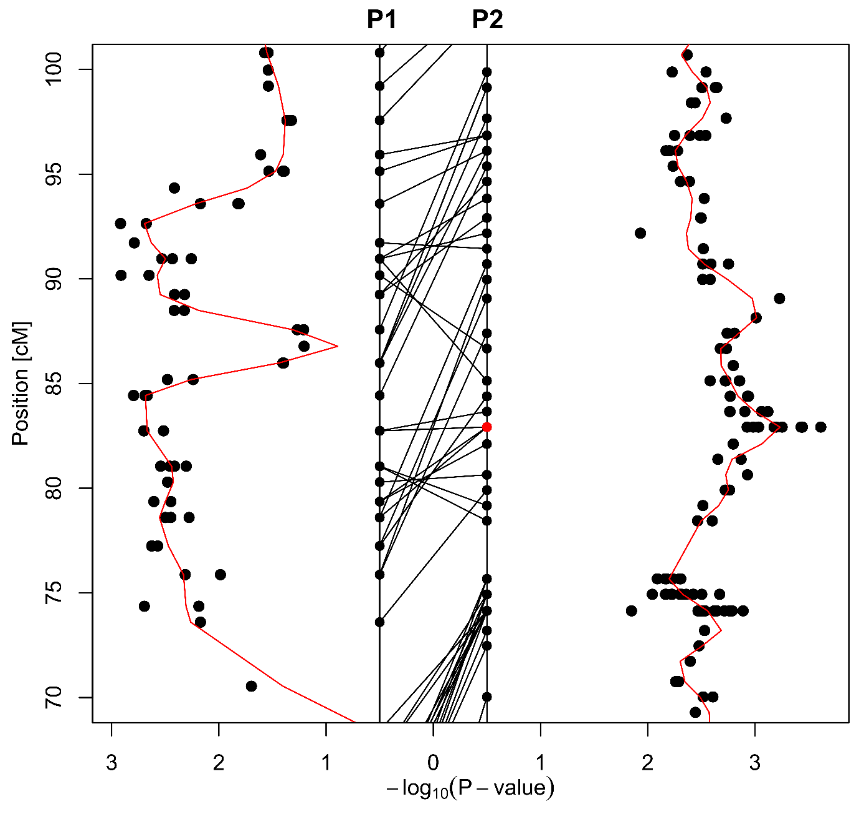


**Figure S5 LOD score and linkage map comparison between P1 (left) and P2 (right) on chromosome 1R**. In the middle of the plot, the markers of both population-specific linkage maps are visualized as dots on two vertical strands with cM distances given on the y-axis. Overlapping markers between both populations are connected by lines. LOD scores (-log10(p-value)) for mapping of leaf rust resistance are plotted to the left and to the right (mirrored x-axis), with the value of zero in the center. The red dot gives the position of the most significant marker (isotig221292) in P2, which was monomorphic in P1.
